# Trends in procedural closure of patent ductus arteriosus among children from 2011 to 2022 in Japan

**DOI:** 10.1111/ped.70388

**Published:** 2026-04-07

**Authors:** Kyosuke Ibi, Yoshihisa Miyamoto, Yuichiro Matsuo, Ryo Inuzuka, Kiyohide Fushimi, Motohiro Kato, Hideo Yasunaga

**Affiliations:** ^1^ Department of Pediatrics, Graduate School of Medicine The University of Tokyo Tokyo Japan; ^2^ Department of Clinical Epidemiology and Health Economics, Graduate School of Medicine The University of Tokyo Tokyo Japan; ^3^ Department of Real‐World Evidence, Graduate School of Medicine The University of Tokyo Tokyo Japan; ^4^ Department of Health Policy and Informatics Institute of Science Tokyo Graduate School Tokyo Japan

**Keywords:** cardiac catheterization, cardiac surgery, congenital heart disease, patent ductus arteriosus, preterm infant

## Abstract

**Background:**

Although surgery was traditionally the primary modality for definitive patent ductus arteriosus (PDA) closure, the indications for transcatheter closure have expanded with device advancements in recent decades. This study aimed to describe the trends in surgical and transcatheter closure for PDA in Japan.

**Methods:**

Patients aged <16 years who underwent procedural PDA closure between April 2011 and March 2023 were identified from the Diagnosis Procedure Combination database in Japan. Considering the redaction of the requirements for the Amplatzer Duct Occluder (age ≥6 months and weight ≥6 kg) in 2016, patients were stratified by this erstwhile weight threshold (6 kg) to evaluate the impact of this regulatory change. We analyzed data from consecutive 2‐year periods. We examined the trend in the proportion of transcatheter closure among all procedural PDA closures using multivariable mixed‐effects logistic regression models.

**Results:**

The study enrolled 5638 patients (3235 weighing <6 kg and 2403 weighing ≥6 kg). The proportion of transcatheter closure increased over time from 13/466 (2.8%) patients in 2011–2012 to 60/431 (13.9%) in 2021–2022 in patients weighing <6 kg (adjusted odds ratio 1.46, 95% confidence interval 1.29–1.67), and from 268/316 (84.8%; 2011–2012) to 392/416 (94.2%; 2021–2022) in patients weighing ≥6 kg (1.56, 1.38–1.76).

**Conclusions:**

Although the proportion of transcatheter closure steadily increased in patients weighing <6 kg, its implementation was slow and surgical closure remained the primary treatment. Transcatheter closure has become the predominant option for patients weighing ≥6 kg.

## INTRODUCTION

Patent ductus arteriosus (PDA) is one of the most prevalent congenital heart diseases (CHDs), with an even greater incidence in preterm infants.^1^ A large, symptomatic, or persistent PDA requires procedural closure, which includes surgical and transcatheter closure. Surgical closure has traditionally been the standard procedure for PDA management. In recent decades, transcatheter closure has been shown to be a less invasive and effective alternative with fewer complications and shorter lengths of hospital stay that yields comparable success rates relative to surgical closure in pediatric patients.^2^, ^3^, ^4^ Transcatheter closure is now the procedure of choice for PDA treatment in adults and children weighing ≥6 kg.^2^, ^4^, ^5^, ^6^ The indications for transcatheter closure have been expanded to smaller infants, concurrent with the innovations in new catheter devices, and a tendency toward transcatheter closure has emerged for procedural PDA closure, even in preterm infants.^7^, ^8^, ^9^ However, international variations persist with respect to the procedural timing and methods for PDA in small infants, particularly preterm infants.^10^, ^11^, ^12^, ^13^

In Japan, the Amplatzer Duct Occluder (ADO) was first approved for use in patients aged ≥6 months or weighing ≥6 kg in 2009, and the indication has expanded after the restrictions on age and weight were lifted in 2016. Subsequently, the ADO‐II received approval for use in patients weighing ≥2.5 kg in 2019, and the Amplatzer Piccolo Occluder (APO) for use in patients weighing ≥700 g in 2020.^14^

Few studies have reported the trends in procedural PDA closure in Japan. Documenting treatment patterns following regulatory changes would be helpful in assessing the real‐world impact of device approval updates and enabling comparisons with the clinical practices in other countries. In this study, we aimed to describe the trends in PDA treatment in Japan, focusing on the comparison between surgical and transcatheter closure using data from a nationwide inpatient database.

## METHODS

### Data source

This nationwide, retrospective cohort study analyzed data extracted from the Japanese Diagnosis Procedure Combination (DPC) database, which includes hospital data on administrative claims and discharge summaries.^15^ More than 1200 acute‐care hospitals voluntarily contribute to this database. The DPC covers approximately 90% of the hospitals with neonatal and pediatric intensive care units in Japan.^16^, ^17^

The DPC database comprehensively stores the following data: unique hospital identifiers; age; sex; gestational age; birth weight; primary diagnoses and comorbidities on admission, complications following admission recorded in Japanese text, International Classification of Diseases, Tenth Revision (ICD‐10) codes, and the original Japanese procedure codes; drugs; length of stay; and discharge status. Previous validation studies have demonstrated good sensitivity and specificity for the diagnostic and procedural records in the DPC database.^18^, ^19^, ^20^

This study was conducted in accordance with the ethical standards of the Institutional and National Research Committee and the 1964 Helsinki Declaration and its later amendments or comparable ethical standards. The Institutional Review Board of the University of Tokyo approved this study (approval number: 3501‐(5) [May 19, 2021]). The requirement for informed consent was waived because the analysis was restricted to anonymized data.

### Study participants

We retrospectively identified patients aged <16 years who were hospitalized under a diagnosis of PDA (ICD‐10 code: Q250, along with the Japanese text data) between April 2011 and March 2023. We included patients with procedural closure (surgical or transcatheter) recorded using the Japanese procedure codes. Because the procedure codes do not distinguish between coil embolization and device occlusion, both procedures were designated as transcatheter closure. We categorized the study chronology into six intervals of 2 consecutive fiscal years each: 2011–2012, 2013–2014, 2015–2016, 2017–2018, 2019–2020, and 2021–2022. To compare surgical and transcatheter closure over a given period, we restricted the study population to patients treated in hospitals that had performed at least one procedural closure within all six intervals. Patients with missing body weight data on admission were excluded. We also categorized the study population into patients weighing <6 kg or ≥6 kg on admission. This cutoff was selected because it was the threshold defining the indication for transcatheter closure using the ADO, which was removed in 2016.

### Definition of variables and study outcomes

We handled multiple episodes of hospitalization in each patient separately to describe the trends in the number of procedural closures. When the same procedure was repeated during a single hospitalization, it was counted as one closure. Gestational age and birth weight were only documented for patient characteristics in patients weighing <6 kg because these data were not available in the database after the neonatal period for numerous patients. We presented the proportion of procedural PDA closure in patients born between 22 and 27, 28 and 31, and 32 and 36 weeks of gestation and low birth weight infants weighing <1000 g, 1000–1499 g, and 1500–2499 g in patients weighing <6 kg. We identified patients with other CHDs using the ICD‐10 and procedure codes. We defined complex chronic conditions (CCCs) using the Pediatric CCC Classification System, version 2, including the neonatal CCC category, if applicable.^21^ The definitions of other CHDs, CCCs, and chromosomal disorders are provided in Table S1. We identified the following supportive therapies performed on the day before the procedure: mechanical ventilation, catecholamine use, and diuretic use. Mechanical ventilation included both invasive and noninvasive ventilatory support. We determined whether intravenous indomethacin and ibuprofen were administered as medical therapy for PDA before the procedure, since they are currently indicated as pharmacotherapy for PDA in preterm infants. Because indomethacin is approved both for the treatment of PDA and the prevention of intraventricular hemorrhage, we could not determine the primary purpose of indomethacin use from the information provided in the database. Ibuprofen has been covered for the treatment of PDA since 2018.

We ascertained the trends in the proportion of transcatheter closure among the total procedural closures during each interval. We also determined the trends in the patients' characteristics and outcomes (length of hospital stay and in‐hospital mortality).

### Statistical analysis

Categorical variables were described as frequencies and percentages, and continuous variables as medians and interquartile ranges. We limited our study to the description of the procedural trends and did not perform statistical tests to evaluate the relationship between the covariates and procedural outcomes of both procedures. The Cochran–Armitage trend test and linear regression analysis were employed to describe the changes in the patient characteristics and proportion of transcatheter closure among the total procedural closures with two‐fiscal‐year intervals. We constructed multivariable mixed‐effects logistic regression models to investigate the independent relationship between the two‐fiscal‐year intervals of admission with procedural selection, accounting for within‐hospital patient clustering.^22^ All models treated hospitals as a random effect. We evaluated the temporal trend by including two‐fiscal‐year intervals as a continuous variable in model 1, adjusting for sex, age at procedure, weight on admission, concomitant CHDs, CCCs, and chromosomal disorders. We further adjusted for gestational age and birth weight in model 2, in addition to the variables adjusted in model 1. Since gestational age and birth weight were missing in approximately half of the patients weighing ≥6 kg, we designated model 1, which excludes these variables, as the primary model. For the subgroup analyses, we described procedural trends in four specific patient groups: patients weighing <2.5 kg on admission, those weighing <1.0 kg on admission, those with other CHDs, and those with chromosomal disorders. We focused on patients with other CHDs and chromosomal disorders because these comorbidities are associated with distinct etiologies and treatment indications for PDA.^3^, ^4^, ^23^ The threshold for significance was set at *p* < 0.05. All statistical analyses were conducted using Stata/SE (version 18.0; StataCorp, College Station, Texas, USA).

## RESULTS

**FIGURE 1:**
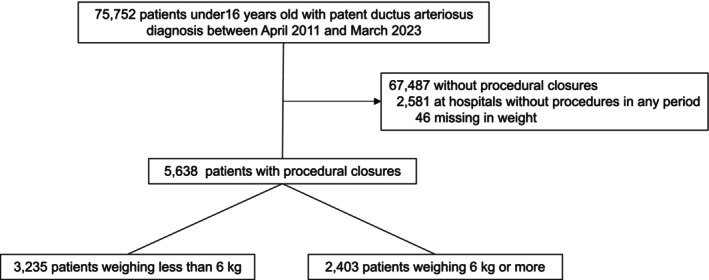
Flow diagram of participant selection.

During the study period, we identified 75,752 patients aged <16 years who were diagnosed with PDA. We excluded 67,487 patients without procedural closure, and 2581 patients treated at 113 hospitals where procedural closures were not performed during all the six 2‐year intervals. We also excluded 46 patients with missing body weight data. We identified 5638 eligible patients across 71 hospitals, of whom 3235 weighed <6 kg and 2403 weighed ≥6 kg (Figure 1). Overall, 3057 surgical and 185 transcatheter closures were performed in patients weighing <6 kg, and 266 surgical and 2137 transcatheter closures in patients weighing ≥6 kg. Seven patients weighing <6 kg underwent both surgical and transcatheter closure during hospitalization. No patients weighing ≥6 kg underwent two different procedures during hospitalization.

### Trends of the patient characteristics in patients weighing <6 kg

In patients weighing <6 kg, the median weight on admission for those who underwent surgical closure was 1.0 kg, and this trend remained unchanged (Table 1). Transcatheter closure was performed predominantly for patients weighing <2.5 kg in 2011–2012; however, after 2013–2014, the majority of patients undergoing this procedure weighed ≥2.5 kg and were several months of age.

**TABLE 1:** Characteristics of patients treated with surgical or transcatheter patent ductus arteriosus closure from 2011 to 2022 in the group weighing <6 kg.

Year	2011–2012		2013–2014		2015–2016		2017–2018		2019–2020		2021–2022		*p* Value	
	Surgery	Catheter	Surgery	Catheter	Surgery	Catheter	Surgery	Catheter	Surgery	Catheter	Surgery	Catheter	Surgery	Catheter
Total	466		629		649		586		481		432		–	
No. of procedures	453	13	604	25	624	25	563	23	442	39	371	61	–	–
No. of hospitals	67	6	69	12	70	11	70	13	68	14	65	16	–	–
Weight on admission, kg	1.0 [0.7–2.6]	1.2 [0.9–2.6]	1.0 [0.7–2.7]	3.1 [1.2–5.0]	1.1 [0.7–2.7]	4.1 [1.5–5.3]	1.0 [0.7–2.6]	4.4 [3.1–5.1]	1.0 [0.7–2.6]	4.7 [3.8–5.3]	1.1 [0.7–2.4]	4.2 [2.4–5.2]	0.27	0.002
Age at procedure, day	22 [12–38]	24 [16–32]	22 [12–35]	60 [20–194]	22 [11–36]	121 [18–188]	21 [12–34]	110 [30–147]	20 [11–33]	131 [60–172]	19 [10–30]	84 [40–134]	<0.001	0.63
Basic characteristics														
Male	210 (46.4%)	7 (53.8%)	254 (42.1%)	9 (36.0%)	262 (42.0%)	7 (28.0%)	246 (43.7%)	6 (26.1%)	204 (46.2%)	16 (41.0%)	165 (44.5%)	20 (33.3%)	0.74	0.59
Gestational age, weeks	26 [24–34]	27 [25–29]	26 [24–35]	30 [29–39]	26 [24–35]	30 [24–38]	25 [24–36]	38 [33–38]	27 [24–36]	37 [36–38]	27 [24–36]	37 [34–38]	0.01	<0.001
22–27	209 (46.1%)	6 (46.2%)	280 (46.4%)	3 (12.0%)	317 (50.8%)	7 (28.0%)	302 (53.6%)	3 (13.0%)	228 (51.6%)	2 (5.1%)	181 (48.8%)	6 (10.0%)	–	–
28–31	35 (7.7%)	3 (23.1%)	53 (8.8%)	7 (28.0%)	59 (9.5%)	3 (12.0%)	43 (7.6%)	1 (4.3%)	37 (8.4%)	1 (2.6%)	37 (10.0%)	2 (3.3%)	–	–
33–36	32 (7.1%)	0 (0.0%)	40 (6.6%)	1 (4.0%)	70 (11.2%)	2 (8.0%)	50 (8.9%)	1 (4.3%)	46 (10.4%)	2 (5.1%)	46 (12.4%)	6 (9.8%)	–	–
Missing	119 (26.3%)	3 (23.1%)	127 (21.0%)	7 (28.0%)	72 (11.5%)	6 (24.0%)	53 (9.4%)	7 (30.4%)	29 (6.6%)	19 (48.7%)	21 (5.7%)	16 (26.7%)	–	–
Birth weight, g	847 [652–1772]	1055 [877–1813]	900 [658–1980]	2382 [1177–2885]	903 [656–2118]	2273 [794–2928]	883 [655–2050]	2673 [2205–3020]	918 [655–2140]	2566 [2090–2827]	932 [662–2020]	2638 [1994–2876]	0.06	<0.001
<1000	258 (57.0%)	5 (38.5%)	327 (54.1%)	3 (12.0%)	326 (52.2%)	7 (28.0%)	308 (54.7%)	3 (13.0%)	235 (53.2%)	2 (5.1%)	193 (52.0%)	6 (10.0%)	–	–
1000–1499	52 (11.5%)	3 (23.1%)	64 (10.6%)	6 (24.0%)	64 (10.3%)	1 (4.0%)	50 (8.9%)	1 (4.3%)	43 (9.7%)	3 (7.7%)	40 (10.8%)	2 (3.3%)	–	–
1500–2499	49 (10.8%)	1 (7.7%)	91 (15.1%)	6 (24.0%)	89 (14.3%)	7 (28.0%)	89 (15.8%)	4 (17.4%)	74 (16.7%)	7 (17.9%)	63 (17.0%)	13 (21.3%)	–	–
Missing	29 (6.4%)	2 (15.4%)	35 (5.8%)	2 (8.0%)	42 (6.7%)	1 (4.0%)	30 (5.3%)	5 (21.7%)	14 (3.2%)	12 (30.8%)	16 (4.3%)	11 (18.3%)	–	–
Other CHDs	44 (9.7%)	0 (0.0%)	57 (9.4%)	2 (8.0%)	84 (13.5%)	3 (12.0%)	52 (9.2%)	2 (8.7%)	58 (13.1%)	2 (5.1%)	57 (15.4%)	2 (3.3%)	0.009	0.49
Complex chronic conditions	74 (16.3%)	0 (0.0%)	112 (18.5%)	3 (12.0%)	124 (19.9%)	7 (28.0%)	134 (23.8%)	3 (13.0%)	98 (22.2%)	8 (20.5%)	85 (22.9%)	11 (18.3%)	0.002	0.28
Chromosomal disorders	30 (6.6%)	1 (7.7%)	46 (7.6%)	4 (16.0%)	73 (11.7%)	5 (20.0%)	56 (9.9%)	7 (30.4%)	45 (10.2%)	12 (30.8%)	38 (10.2%)	17 (28.3%)	0.03	0.054
Treatments and outcomes														
Mechanical ventilation	334 (73.7%)	8 (61.5%)	453 (75.0%)	13 (52.0%)	461 (73.9%)	12 (48.0%)	424 (75.3%)	7 (30.4%)	346 (78.3%)	6 (15.4%)	305 (82.2%)	18 (30.0%)	0.003	<0.009
Catecholamine	242 (53.4%)	4 (30.8%)	347 (57.5%)	6 (24.0%)	355 (56.9%)	10 (40.0%)	275 (48.8%)	5 (21.7%)	222 (50.2%)	3 (7.7%)	210 (56.6%)	11 (18.3%)	0.26	0.04
Diuretics	271 (59.8%)	6 (46.2%)	390 (64.6%)	17 (68.0%)	380 (60.9%)	13 (52.0%)	345 (61.3%)	11 (47.8%)	274 (62.0%)	21 (53.8%)	233 (62.8%)	34 (56.7%)	0.82	0.94
Indomethacin or Ibuprofen	261 (57.6%)	9 (69.2%)	363 (60.1%)	10 (40.0%)	312 (50.0%)	7 (28.0%)	302 (53.6%)	2 (8.7%)	262 (59.3%)	5 (12.8%)	218 (58.8%)	8 (13.3%)	0.007	<0.001
Length of stay, days	99 [23–140]	115 [30–127]	96 [27–140]	81 [10–133]	85 [20–131]	15 [5–139]	99 [26–145]	5 [4–33]	100 [26–148]	4 [4–12]	95 [24–147]	5 [4–25]	0.31	<0.001
In‐hospital mortality (Total)	18 (3.9%)		30 (4.8%)		28 (4.3%)		29 (4.9%)		16 (3.3%)		21 (4.9%)		0.92	
In‐hospital mortality	17 (3.8%)	1 (7.7%)	30 (5.0%)	0 (0.0%)	28 (4.5%)	0 (0.0%)	29 (5.2%)	0 (0.0%)	16 (3.6%)	0 (0.0%)	20 (5.4%)	1 (1.7%)	0.63	0.52

*Note*: Data are presented as *n* (%) or median [interquartile range].

Abbreviation: CHD: congenital heart disease.

Extremely preterm and extremely low birth weight infants accounted for approximately 50% of patients who underwent surgical closure throughout the study period. Meanwhile, the proportion of preterm infants who underwent transcatheter closure plummeted from 69.3% in 2011–2012 to 23.1% in 2021–2022.

Surgical closure was increasingly selected in patients with other comorbid CHDs, patients with CCCs, and patients with chromosomal disorders.

Supportive therapy before the procedure was required in over 50% of patients undergoing surgical closure, and this trend became more apparent in recent times. Supportive therapies were not frequently required in patients undergoing transcatheter closure, with a decrease in mechanical ventilation and catecholamine use over time.

The length of hospital stay in patients who underwent transcatheter closure decreased from 2015 to 2016. The overall mortality rate remained stable throughout this period. The in‐hospital mortality was relatively lower with transcatheter closure than with surgical closure.

### Trends in the characteristics of patients weighing ≥6 kg

In patients weighing ≥6 kg, patients undergoing surgical closure were treated at a median weight of 7.9 kg and median age of 21 days, while patients undergoing transcatheter closure were treated at a median weight of 13.3 kg and median age of 103 days (Table 2).

**TABLE 2:** Characteristics of patients treated with surgical or transcatheter patent ductus arteriosus closure: From 2011 to 2022 in the group weighing ≥6 kg.

	2011–2012		2013–2014		2015–2016		2017–2018		2019–2020		2021–2022		*p* Value	
	Surgery	Catheter	Surgery	Catheter	Surgery	Catheter	Surgery	Catheter	Surgery	Catheter	Surgery	Catheter	Surgery	Catheter
Total	316		422		432		419		398		416		–	
No. of procedures	48	268	63	359	57	375	42	377	32	366	24	392	–	–
No. of hospitals	30	32	33	38	31	43	26	40	20	36	14	37	–	–
Weight on admission, kg	8.5 [7.0–12.2]	13.9 [10.6–18.7]	8.1 [6.9–10.1]	13.0 [9.6–17.4]	8.1 [7.0–9.1]	12.3 [9.5–17.4]	7.8 [6.8–9.4]	14.6 [10.5–20.0]	7.8 [6.6–9.3]	13.3 [9.7–18.9]	7.5 [6.6–9.0]	13.2 [9.8–18.1]	0.01	0.22
Age at procedure, month	15 [9–29]	42 [24–68]	13 [6–23]	37 [20–65]	11 [7–18]	38 [20–63]	13 [8–17]	44 [22–76]	11 [6–18]	40 [19–72]	12 [6–17]	40 [20–68]	0.02	0.32
Basic characteristics														
Male	20 (41.7%)	80 (29.9%)	25 (39.7%)	109 (30.4%)	24 (42.1%)	129 (34.4%)	20 (47.6%)	130 (34.5%)	15 (46.9%)	139 (38.0%)	9 (37.5%)	136 (34.7%)	0.75	0.03
Other CHDs	2 (4.2%)	4 (1.5%)	10 (15.9%)	7 (1.9%)	9 (15.8%)	11 (2.9%)	6 (14.3%)	7 (1.9%)	9 (28.1%)	5 (1.4%)	6 (25.0%)	8 (2.0%)	0.007	0.90
Complex chronic conditions	2 (4.2%)	2 (0.7%)	5 (7.9%)	7 (1.9%)	3 (5.3%)	6 (1.6%)	7 (16.7%)	6 (1.6%)	5 (15.6%)	13 (3.6%)	5 (20.8%)	11 (2.8%)	0.006	0.02
Chromosomal disorders	3 (6.2%)	11 (4.1%)	7 (11.1%)	27 (7.5%)	5 (8.8%)	29 (7.7%)	7 (16.7%)	26 (6.9%)	7 (21.9%)	28 (7.7%)	6 (25.0%)	34 (8.7%)	0.006	0.08
Treatments and outcomes														
Mechanical ventilation	0 (0.0%)	0 (0.0%)	0 (0.0%)	2 (0.6%)	1 (1.8%)	2 (0.5%)	0 (0.0%)	4 (1.1%)	1 (3.1%)	4 (1.1%)	0 (0.0%)	2 (0.5%)	0.44	0.95
Catecholamine	7 (14.6%)	0 (0.0%)	14 (22.2%)	1 (0.3%)	12 (21.1%)	1 (0.3%)	9 (21.4%)	1 (0.3%)	10 (31.2%)	1 (0.3%)	7 (29.2%)	2 (0.5%)	0.09	0.31
Diuretics	13 (27.1%)	10 (3.7%)	19 (30.2%)	23 (6.4%)	20 (35.1%)	30 (8.0%)	19 (45.2%)	26 (6.9%)	20 (62.5%)	24 (6.6%)	10 (41.7%)	20 (5.1%)	0.003	0.81
Length of stay, days	13 [10–17]	4 [3–5]	13 [9–17]	4 [3–5]	11 [8–15]	4 [3–5]	13 [11–17]	4 [3–5]	13 [11–24]	3 [3–4]	14 [5–20]	3 [2–4]	0.051	0.04
In‐hospital mortality (Total)	0 (0.0%)		0 (0.0%)		1 (0.2%)		0 (0.0%)		0 (0.0%)		0 (0.0%)		0.72	
In‐hospital mortality	0 (0.0%)	0 (0.0%)	0 (0.0%)	0 (0.0%)	1 (1.8%)	0 (0.0%)	0 (0.0%)	0 (0.0%)	0 (0.0%)	0 (0.0%)	0 (0.0%)	0 (0.0%)	0.96	NA

*Note*: Data are presented as *n* (%) or median [interquartile range].

Abbreviation: CHD, congenital heart disease.

The proportion of patients with other comorbid CHDs, CCCs, and chromosomal disorders increased among those who underwent surgical closure over time. Approximately one‐fourth of patients with PDA had any one of these complications in 2021–2022. Most patients who underwent transcatheter closure did not have other CHDs, CCCs, or chromosomal disorders throughout this period. Mechanical ventilation was rarely needed in either procedure. Catecholamines and diuretics were administered more frequently to patients undergoing surgical closure, and the proportion of use increased slightly from 14.6% and 27.1% in 2011–2012 to 29.2% and 41.7% in 2021–2022, respectively.

The median lengths of hospital stay were 13 and 4 days for surgical and transcatheter closure, respectively. The in‐hospital mortality rates were extremely low in both groups.

### Trends in the proportions of procedural closures

**FIGURE 2:**
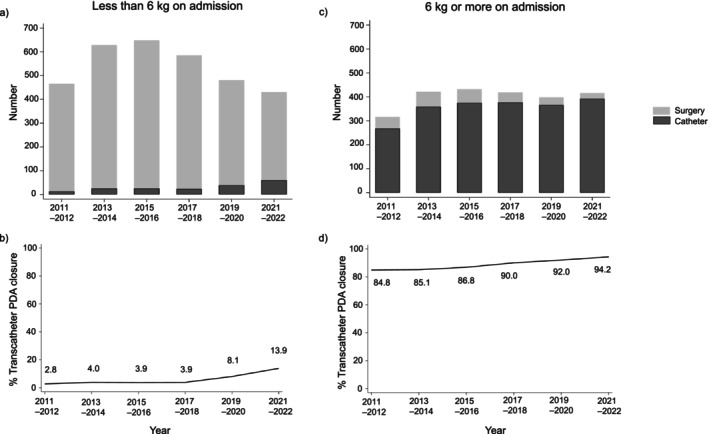
Trends in surgical and transcatheter closures. (a) Trend in the number of surgical and transcatheter closures in patients weighing <6 kg. (b) Proportion of transcatheter closure among the total procedural closures in patients weighing <6 kg. (c) Trend in the number of surgical and transcatheter closures in patients weighing ≥6 kg. (d) Proportion of transcatheter closure among the total procedural closures in patients weighing ≥6 kg.

**FIGURE 3:**
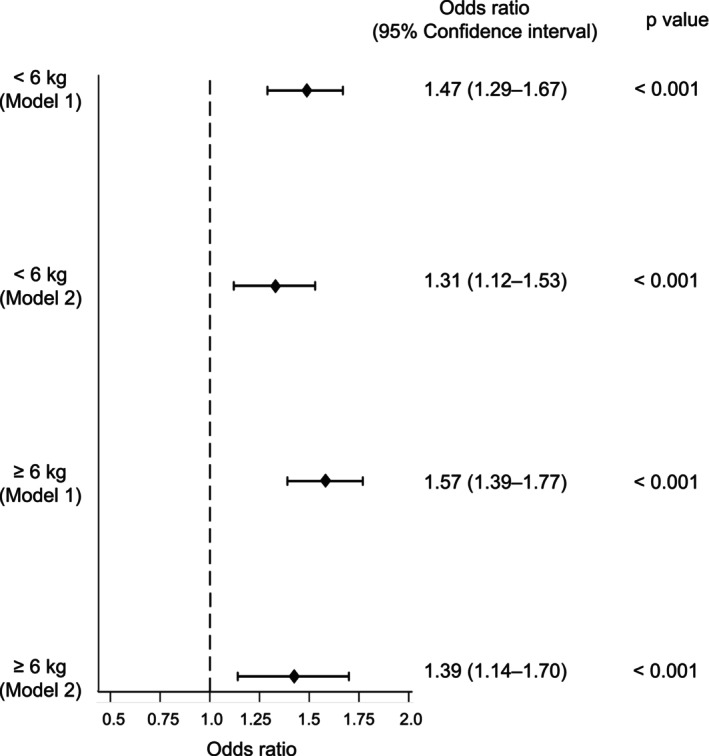
Trends in surgical and transcatheter closures. Multivariable mixed‐effects logistic regression models showing the relationship between the two‐fiscal‐year intervals of admission with transcatheter closure in procedures. Model 1: Adjusted for weight on admission, sex, age at procedure, concomitant congenital heart diseases, complex chronic conditions, and chromosomal disorders. Model 2: Adjusted for variables in model 1 plus gestational age and birth weight.

In 2015–2016, the total number of procedural closures peaked, and decreased gradually in patients weighing <6 kg, but remained constant in patients weighing ≥6 kg (Figure 2). In patients weighing <6 kg, the proportion of transcatheter closure rose significantly from 2.8% (13/466) in 2011–2012 to 13.9% (60/431) in 2021–2022 (P for trend <0.001). The multivariable‐adjusted model showed that the likelihood of selecting transcatheter closure increased significantly over time [model 1: adjusted odds ratio for two‐fiscal‐year intervals (aOR) 1.47, 95% confidence interval (CI) 1.29–1.67; and model 2: aOR 1.31, 95% CI 1.12–1.53] (Figure 3). In patients weighing ≥6 kg, the proportion of transcatheter closure showed further elevation, that is, 84.8% (268/316) in 2011–2012 and 94.2% (392/416) in 2021–2022 (P for trend <0.001). After adjusting for patient characteristics and hospitals, the likelihood of selecting transcatheter closure among the total procedural closures increased over time (model 1: aOR 1.57, 95% CI 1.37–1.77; and model 2: aOR 1.39, 95% CI 1.14–1.70).

### Trends in procedural closure in the subgroups

A total of 2203 surgical and 53 transcatheter, and 1535 surgical and 24 transcatheter closures were performed in patients weighing <2.5 kg and <1.0 kg on admission, respectively (Figure S1). The number of procedural closures peaked in 2013–2014 and 2015–2016. The proportion of transcatheter closure has not changed from 2.6% (9/340) in 2011–2012 to 5.7% (17/280) in 2021–2022 for patients weighing <2.5 kg (P for trend = 0.28), and from 2.0% (5/245) in 2011–2012 to 3.3% (6/182) in 2021–2022 for patients weighing <1.0 kg (P for trend = 0.91). During the study period, patients with other CHDs and chromosomal disorders received 394 and 323 surgical procedures, and 52 and 201 transcatheter closures, respectively. The total number of procedural closures increased until 2015–2016 but stabilized in recent periods. In patients with other CHDs and chromosomal disorders, the trend in the proportion of transcatheter closure changed from 6.1% (3/49) and 26.7% (12/45) in 2011–2012 to 13.7% (10/73) and 53.7% (51/95) in 2021–2022 (P for trend = 0.61 and <0.001; Figure S2).

## DISCUSSION

We observed distinct trends in the choice of procedural PDA closure according to patient weight, using nationally representative data from Japanese patients hospitalized between 2011 and 2022. Among patients weighing <6 kg, the proportion of transcatheter closure increased from 2015 – 2016 onwards, especially in patients aged several months who did not require supportive therapies. Surgical closure was performed more commonly in low birth weight infants during the neonatal period and often involved more preoperative supportive therapies than transcatheter closure.

For patients weighing ≥6 kg, transcatheter closure was the primary approach throughout the study period. The proportions of underlying conditions such as other CHDs, CCCs, and chromosomal disorders and the requirement of preoperative supportive therapies were higher in patients receiving surgical closure.

Moreover, the increase in the proportion of transcatheter closure in patients weighing <2.5 kg was not substantial, and surgical closure was predominant throughout the study period.

### Procedures for PDA in Japan

The remarkable increase in the proportion of transcatheter closure after 2016 in patients weighing <6 kg may reflect the expanded application of catheter devices. In 2011–2012, off‐label transcatheter closure was performed in a limited number of patients weighing <2.5 kg. The gradual increase in the number of patients weighing ≥2.5 kg who underwent transcatheter closure can be attributed to the expansion of the indications for ADO in 2016 and the approval of the ADO‐II in 2019.^14^ However, the implementation of transcatheter closure was gradual, even after the approval of these new devices. For procedural PDA closure in patients weighing <6 kg in Japan, a tendency toward surgical closure for lower weight patients requiring intensive care was apparent, and transcatheter closure for patients with elective procedures aged several months. Similarly, in other countries, surgical closure tended to be preferred for more severely ill patients.^24^, ^25^ This preference may reflect several clinical considerations: ductal morphology less amendable to device closure in preterm infants, contraindications to procedural heparin use, and the risks associated with transporting critically ill patients to catheterization facilities. The recent shortening of hospitalization periods observed for patients undergoing transcatheter closures may reflect an increase in patients admitted for elective procedures. The lack of increase in transcatheter closures among patients with other CHDs may be related to PDA morphology in these patients and the necessity for treating concomitant conditions with surgical procedures. In‐hospital mortality remained low even after the increase in the use of transcatheter closure, suggesting safe implementation of these procedures. The proportion of transcatheter closure increased slightly after the APO received approval. However, because APO was approved in Japan only recently in 2020, continuous monitoring is needed since post‐approval experience is limited.

For patients weighing ≥6 kg, transcatheter closure was the mainstay throughout the study period, yielding favorable outcomes. However, surgical closure was needed for a limited number of patients, some of whom had other comorbid CHDs, CCCs, and chromosomal disorders. The number of transcatheter closures increased slightly for patients with chromosomal disorders, presumably because of the expanded range of device options available.^26^, ^27^

### Comparison of the procedural trends with other countries

In patients weighing ≥6 kg, the wider application of transcatheter closure compared with surgical closure in Japan was consistent with international trends.^4^, ^6^ This may reflect the international consensus that prioritizes transcatheter closure as the standard treatment for patients weighing ≥6 kg.^2^, ^4^, ^5^, ^6^

Conversely, in patients weighing <6 kg, the international consensus for the optimal procedures for PDA remains elusive.^28^, ^29^ For example, a nationwide study from the United Kingdom and Ireland conducted in 2015 reported that the proportion of transcatheter PDA closure for patients aged 1–12 months was 36.9% (107/290), whereas that for patients aged <1 month was only 1.3% (1/75).^30^ Meanwhile, although the APO was approved in 2019 in the United States, transcatheter closure found rapid widespread application and surpassed surgical closure even in extremely preterm or extremely low birth weight infants in the period around 2019 to 2021.^24^, ^25^, ^31^, ^32^, ^33^ The trend in the rapid increase in the proportion of transcatheter closures in the US contrasts with the more gradual increase in Japan. The latter may be partly attributed to the Japanese guideline for the safe implementation of APO published in 2019. The guideline mandates stringent criteria for catheterization centers and operators and recommends that surgical closure should be prioritized for patients weighing <1 kg.^14^ In contrast, the current North American guideline suggests that transcatheter closure may be the preferred approach over surgical closure in preterm infants if sufficient institutional expertise is available and the patient characteristics are suitable.^34^ The difference in institutional certification and specialist qualifications may be one factor responsible for the gap between countries in the selection of the procedures in small infants.

In addition to these guidelines, regional differences may also be ascribed to diverse healthcare system structures, resource availability, regulatory environments, and training infrastructures across regions, as reported for other CHDs.^35^, ^36^, ^37^, ^38^ Continued evaluations are warranted to determine suitable interventions for PDA in patients weighing <6 kg.

### Limitations

This study had some limitations associated with the properties of the DPC database. First, the DPC database lacks information on clinical symptoms, radiological and echocardiographic findings, detailed respiratory mode, drug dosage, devices used for the procedures, and procedural success or failure for each PDA closure. The presence of a procedure code does not necessarily mean that the procedure was successful. Second, reinterventions due to recanalization after discharge cannot be reliably traced back to the index admission due to the limitation of the DPC database with respect to longitudinal linkage. Third, to eliminate the influence of differences in the timing of DPC enrollment among hospitals, we restricted the study population to patients treated in hospitals where procedural closures were performed consecutively throughout the study period. The selection of facilities included in the study may have influenced the results. Fourth, we could not obtain information on procedural success or failure and outcomes after discharge. Therefore, the results of the present study should be interpreted as temporal trends in procedural selection rather than in terms of procedural success, complication rates, or quality of care.

## CONCLUSIONS

Based on the analysis of a nationwide database from 2011 to 2022, we found that the proportion of transcatheter closure increased in patients weighing <6 kg, reflecting the expansion of therapeutic indications for catheter devices in Japan; however, the expansion of the use of transcatheter closure was slow even after the approval of these new devices, especially in patients weighing <2.5 kg. Our data will be fundamental for exploring optimal individualized strategies for PDA closure.

## AUTHOR CONTRIBUTIONS

K.I., Y.M. (Yoshihisa Miyamoto), and H.Y. designed the study; K.I., and Y.M. (Yoshihisa Miyamoto) collected and analyzed data, and wrote the manuscript; Y.M. (Yuichiro Matsuo), R.I., K.F, M.K., and H.Y. gave technical support and conceptual advice. All authors read and approved the final manuscript.

## INFORMED CONSENT

The requirement for informed consent was waived because the analysis was restricted to anonymized data.

## FUNDING INFORMATION

This work was supported by grants conferred by the Ministry of Health, Labour and Welfare, Japan (23AA2003 and 24AA2006).

## CONFLICT OF INTEREST STATEMENT

Y.M. (Yoshihisa Miyamoto) has an academic affiliation with the Department of Real‐World Evidence, which is a cooperative program between the University of Tokyo and DeSC Healthcare. K.I., Y.M. (Yuichiro Matsuo), R.I., K.F., M.K., and H.Y. declares no conflict of Interest to disclose.

## Supporting information


Appendix S1.

## Data Availability

The datasets analyzed during the current study are not publicly available due to contracts with the hospitals providing data to the database.
