# Supplementary material for: Trends in procedural closure of patent ductus arteriosus among children from 2011 to 2022 in Japan
Source: Pediatr Int. 2026 Apr 7;68(1):e70388. doi: 10.1111/ped.70388 (PMC13054630; doi:10.1111/ped.70388)
Supplement: Supplementary file 1 — Appendix S1. [file PED-68-e70388-s001.docx]

**Index: Supplemental Materials**

**Supplemental Table 1.** Categories of the underlying conditions according to the ICD-10 Diagnostic and Procedure Codes

**Supplemental Figure 1.** Trends in surgical and transcatheter closure in patients weighing < 2.5 kg and < 1.0 kg

**Supplemental Figure 2.** Trends in surgical and transcatheter closure in patients with congenital heart diseases and chromosomal disorders

**Supplemental Table 1.** **Categories of the underlying conditions according to the ICD-10 Diagnostic and Procedure Codes**

| **Categories** | **ICD-10** |
| --- | --- |
| **Other congenital heart diseases** Q20, Q212-Q219, Q220, Q223, Q225, Q226, Q234, Q246, Q248, Q249, Q251  **(including procedure codes)** Q252, Q254, Q255, Q257–Q259, Q260, Q262-Q269, Q289  K554, K554-2, K555, K555-2, K555-3, K556, K556-2, K557, K557-2, K557-3  K557-4, K558, K559, K559-2, K559-3, K563, K564K565, K566, K567, K567-2  K568, K569, K570, K570-2, K570-3, K570-4, K571, K572, K573, K574  K574-3, K574-4, K575, K576, K577, K578, K579, K579-2, K580, K581, K582  　　　　　　　　　　　　　　　　　K583, K584, K585, K586, K587, K588, K589, K590, K591 | |
| **Comorbidities**  **・Neurologic** G70, G71, G910, G911, G918, G919, Q00-Q07  **・Respiratory** Q30–Q34  **・Gastrointestinal** K44, Q39–Q44, Q790-795  **・Renal, Urologic or Genital** Q60–Q64  **・Hematologic or Immunologic** D55–58, D64, D66, D67, D70, D71, D720, D80–84  **・Endocrine** E00, E23, E25, E70–72, E74, E75–E80, E84, Q77–78  **・Congenital infection** A50, P35, P371  **・Deformity** Q71–76, Q86, Q870–872, Q878, Q897, Q898, Q899 | |
| **Chromosomal disorders** Q90–99 | |

ICD-10: International Classification of Diseases, Tenth Revision

**Supplemental Figure 1. Trends in surgical and transcatheter closure in patients weighing < 2.5 kg and < 1.0 kg**

**
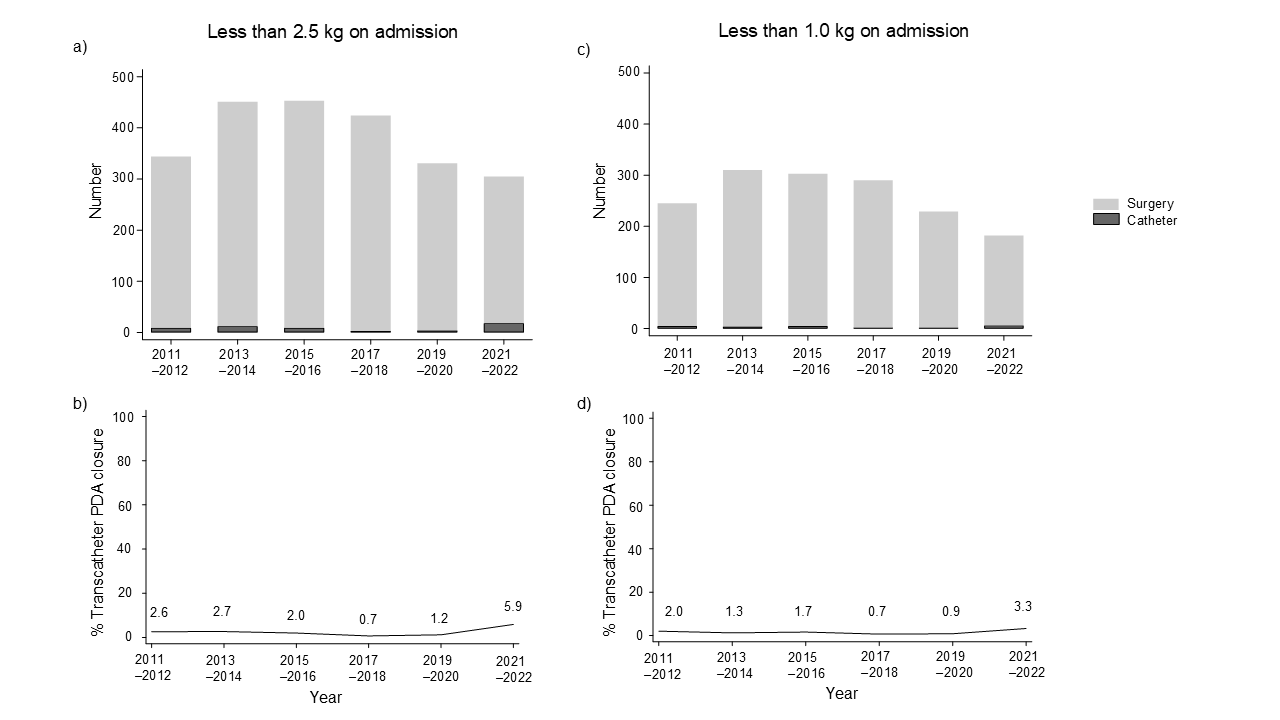
**

a) Trend in the number of surgical and transcatheter closures in patients weighing < 2.5 kg

b) Proportion of transcatheter closure among the total procedural closures in patients weighing < 2.5 kg

c) Trend in the number of surgical and transcatheter closures in patients weighing < 1.0 kg

d) Proportion of transcatheter closure among the total procedural closures in patients weighing < 1.0 kg

**Supplemental Figure 2. Trends in surgical and transcatheter closure in patients with other congenital heart diseases and chromosomal disorders**


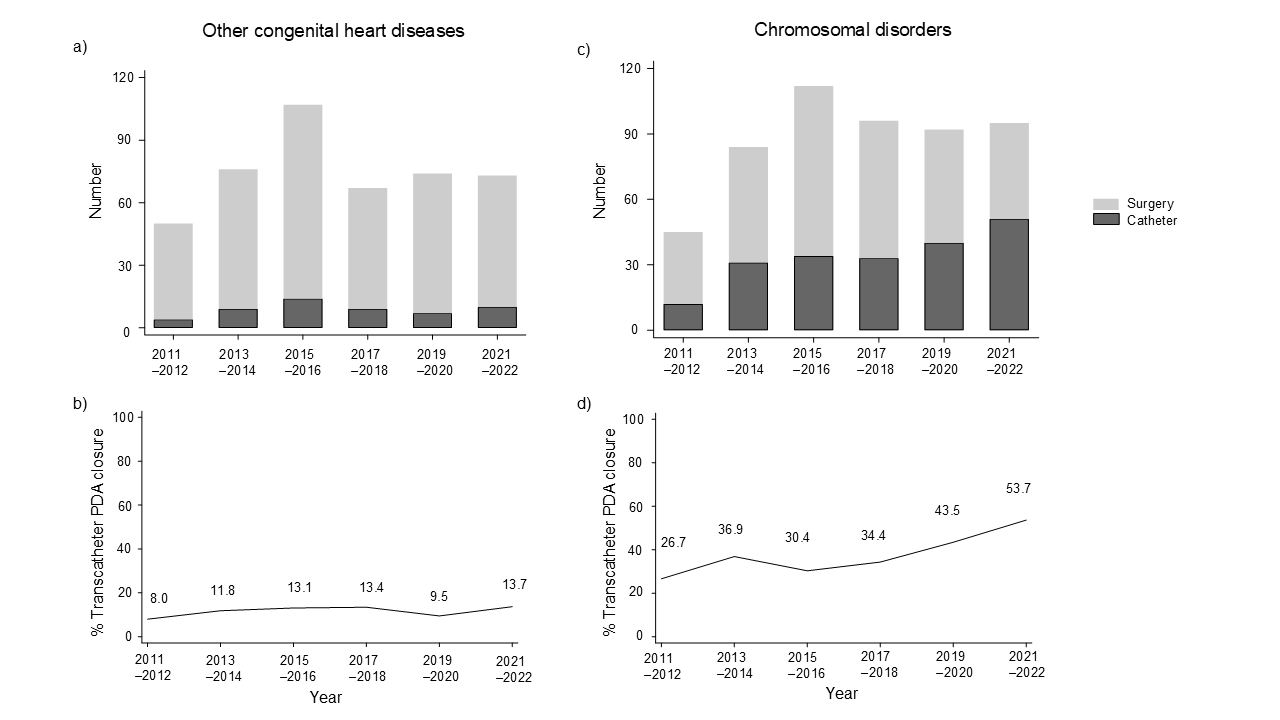


a) Trend in the number of surgical and transcatheter closures in patients with other congenital heart diseases

b) Proportion of transcatheter closure among the total procedural closures in patients with other congenital heart diseases

c) Trend in the number of surgical and transcatheter closures in patients with chromosomal disorders

d) Proportion of transcatheter closure among the total procedural closures in patients with chromosomal disorders
